# A147 “ALREADY GLUTEN-FREE”: A CASE REPORT AND INTERNAL REVIEW OF CHALLENGES IN THE DIAGNOSTIC WORK-UP FOR CHILDREN WITH CELIAC DISEASE

**DOI:** 10.1093/jcag/gwac036.147

**Published:** 2023-03-07

**Authors:** N Lepore, M Zachos, J Dowhanjk

**Affiliations:** Pediatric Gastroenterology, McMaster University, Hamilton, Canada

## Abstract

**Background:**

A single positive tissue transglutaminase IgA (TTG) result does not diagnose celiac disease (CeD). Starting a gluten free diet (GFD) without the proper approach to diagnosis can result in misdiagnosis and inappropriate lifelong gluten restriction. *Helicobacter pylori* (*H. pylori*) infection can be a mimicker of CeD symptomatically, biochemically, and histologically.

**Purpose:**

We report a case of a patient referred for CeD with a false-positive TTG and severe iron deficiency anemia who had started a GFD at the time of referral.

**Method:**

In addition to the case review, we reviewed referrals for possible CeD from June 1 to September 1, 2022 at McMaster Children’s Hospital to identify the proportion of patients started on a partial or full GFD before their initial gastroenterology consultation.

**Result(s):**

**Case Report:**

A 10-year-old female was referred for a positive TTG result 6x the upper limit of normal (72 U/mL; ULN 12U/mL) and iron deficiency anemia with a hemoglobin (Hb) of 85 and MCV of 62. She had been informed her blood work was diagnostic for CeD and to start a GFD prior to referral to Pediatric Gastroenterology. At the time of consultation she had symptoms of GERD, diarrhea, and epigastric pain. Following her consultation, she started a 6-month gluten challenge to proceed to endoscopy. During this time her TTG normalized (2.7 U/ml), however her iron deficiency anemia persisted and was refractory to iron supplementation (Hb 90, MCV 65.9). Endoscopy revealed flattened mucosa in D2 but was otherwise normal. Villous blunting in D1 with severe lymphoplasmacytic gastritis in the body with microabscesses was seen on biopsies. *H. pylori* was negative on Warthin-Starry staining and the pathologist reported a Marsh stage of 3a. Despite the villous blunting, due to the normalized TTG and ongoing epigastric pain, anemia, and presence of lymphoid follicles, a carbon-14 urea breath test was completed which was positive for *H. pylori* (count rate 286 dpm, normal < 50 dpm).

The patient received treatment for *H. pylori* infection for 14 days with 8 week follow-up revealing a negative breath test. There was complete resolution of symptoms, normalization of laboratory results (Hb 143, MCV 82), and negative TTG at 6 months.

**Quality improvement review:**

On internal review of the patients seen in celiac clinic as new referrals from June 1 to September 1 2022, 22/55 patients (42%) had already started a full or partial GFD, therefore requiring gluten challenge and delay in diagnostic investigations.

**Conclusion(s):**

This case demonstrates the importance of following confirmational testing pathways for CeD. It highlights the role of considering potential causes of false positive TTG and the differential diagnosis of CeD such as *H. pylori*, NSAIDs, and Crohn’s disease. The identification of 42% of patients referred for suspected CeD who were already on a GFD, suggests a need for improved education of primary care providers to prevent misdiagnosis and unnecessary life-long gluten elimination diets in children.

**Please acknowledge all funding agencies by checking the applicable boxes below:**

None

**Disclosure of Interest:**

None Declared

